# A metabolic exploration of the protective effect of *Ligusticum wallichii* on IL-1β-injured mouse chondrocytes

**DOI:** 10.1186/s13020-020-0295-0

**Published:** 2020-02-01

**Authors:** Zhiqiang Wei, Chunjiao Dong, Liping Guan, Yafei Wang, Jianghai Huang, Xinzhu Wen

**Affiliations:** 1 Orthopaedics Department, Dongfang Hospital, Beijing University of Chinese Medicine, Bejing, 100078 China; 2 Cardiology & Neurology Department, Beijing TongRen Tang Traditional Chinese Medicine Hospital, Bejing, 100051 China; 3 Intensive Care Unit, Huimin Hospital of Beijing, Bejing, 100013 China

**Keywords:** Osteoarthritis, Chondrocytes, *Ligusticum wallichii*, IL-1β, Metabolic pathways

## Abstract

**Background:**

Osteoarthritis (OA) is a metabolic disorder and able to be relieved by traditional Chinese medicines. However, the effect of *Ligusticum wallichii* on OA is unknown.

**Methods:**

Cytokine IL-1β and *L. wallichii* extracts were used to stimulate the primary mouse chondrocytes. MTT assay was used to measure the cell viability. The mRNA and protein level of each gene were test by qRT-PCR and western blotting, respectively. The rate of apoptotic cell was measured by flow cytometry. GC/MS-based metabolomics was utilized to characterize the variation of metabolome.

**Results:**

Here, we found that *L. wallichii* attenuated the IL-1β-induced apoptosis, inflammatory response, and extracellular matrix (ECM) degradation in mouse chondrocytes. Then we used GC/MS-based metabolomics to characterize the variation of metabolomes. The established metabolic profile of mouse chondrocytes showed that the abundance of most metabolites (*n *= 40) altered by IL-1β stimulation could be repressed by *L. wallichii* treatment. Multivariate data analysis identified that cholesterol, linoleic acid, hexadecandioic acid, proline, l-valine, l-leucine, pyruvate, palmitic acid, and proline are the most key biomarkers for understanding the metabolic role of *L. wallichii* in IL-1β-treated chondrocytes. Further pathway analysis using these metabolites enriched fourteen metabolic pathways, which were dramatically changed in IL-1β-treated chondrocytes and capable of being reprogrammed by *L. wallichii* incubation. These enriched pathways were involved in carbon metabolisms, fatty acid biosynthesis, and amino acid metabolisms.

**Conclusions:**

These findings provide potential clues that metabolic strategies are linked to protective mechanisms of *L. wallichii* treatment in IL-1β-stimulated chondrocytes and emphasize the importance of metabolic strategies against inflammatory responses in OA development.

## Background

Osteoarthritis (OA), a chronic and degenerative joint disease that is appreciated to involve low-grade inflammation and characterizes by the progressive deterioration of articular cartilage, osteophyte formation, subchondral sclerosis, matrix degradation and matrix synthesis imbalance, is one of the most expensive and disabling forms of arthritis, being more widespread than rheumatoid arthritis or other arthritic diseases and representing a major burden of public health [1–4]. In OA, this low-grade inflammation causes a hypoxia condition and resulting metabolic shift in energy metabolism from a resting regulatory state to an extremely metabolically active state to sustain energy homeostasis and promote cell survival, thereby confining the metabolism of pyruvate by the citrate cycle in mitochondria during oxidative phosphorylation and impacting the metabolic flow of chondrocytes and other cells localized in articular cartilage [5–8]. Since several aberrant metabolisms are explored in OA, the increasing and overwhelming evidence indicates that OA is a metabolic disorder [9–11]. Controlling and manipulating cellular metabolism, therefore, has been considered as a realistic and achievable approach for preventing and treating OA.

In past, a traditional Chinese herbal medicine, *Ligusticum wallichii* (family Umbelliferae), is commonly used with other herbs to provide therapeutic intervention in cardiovascular and neurovascular disorders as this herb has an intense ability to repress the muscle contractions and low systemic blood pressure [12]. The new function of *L. wallichii* is capable of protecting host cells against hypoxia-induced injury likely through improving the antioxidant status and immunity profile [13–15]. Since the high inter-relationship between inflammation and hypoxia, we presume that *L. wallichii* may also be benefit for the cells upon OA inflammatory condition. Thus, the first aim of this study is to evaluate the protective effect of *L. wallichii* on cells (for instance, chondrocytes) under inflammatory condition. Then GC/MS-based metabolomics was used to explore the valuable metabolic processes, crucial biomarkers responsible for metabolic characteristics, and metabolic mechanisms.

## Materials and methods

### Chondrocyte isolation and culture

The female C57BL/6 mice were obtained according to internationally accepted principles for laboratory animal use. All work was conducted in strict accordance with the recommendations in the Guide for the Care and Use of Laboratory Animals of the National Institutes of Health. In brief, the mice were euthanized and sterilized, and then the knee joint was collected. After stripping the epidermis, the knee joint was immersed in PBS, cut into slices (2–4 mm thick) and trypsinsied (0.25%) for 30 min. The trypsin-contained supernatant was then removed and chondrocytes were obtained by digesting with 0.5% collagenase type II for 3 h. The chondrocyte suspension was maintained in fresh 10% FBS, high glucose DMEM medium (Hyclone) at 37 °C with 5% CO_2_ atmosphere in a humidified incubator till usage.

### Preparation of *Ligusticum wallichii* extracts

A batch of 50 g of the *L. wallichii* powder was placed in the Soxhlet extractor with 1000 ml flask and continuously extracted using deionized water at the boiling point from 2 to 24 h. All extracts were collected, filtered, concentrated, dried and weighed. *L. wallichii* extracts were prepared in DMSO in 20 mg/ml stock solution and preserved at 4 °C.

### IL-1β and *L. wallichii* treatments

To mimic the OA inflammatory condition, IL-1β was chosen to treat mouse chondrocytes directly. For IL-1β stimulation, 5 × 10^5^ cells were seeded in 6-well plate and exposed to 2 ml medium (1% FBS) containing 10 ng/ml IL-1β (Peprotech) [16]. To investigate the effect of *L. wallichii* alone on mouse chondrocytes, 100, 200, or 400 μg/ml of was *L. wallichii* used for 12, 24 or 48 h. To investigate the effect of *L. wallichii* on IL-1β-injured mouse chondrocytes, cells were treated with IL-1β and either 100 or 200 μg/ml of *L. wallichii* for 12, 24 or 48 h.

### MTT cell viability assay

The effect of *L. wallichii* extraction on improving the proliferation of IL-1β-injured mouse chondrocytes was examined by MTT assay. 2 mg/ml of MTT solution was added to each well and incubated for 3 h at 37 °C. The medium was removed and the blue formazan crystals were dissolved in 200 μl of DMSO and 25 μl Sorenson buffer. The absorbance was recorded in a plate reader (Biotek) at 570 nm. Each experiment was repeated in a triplicate.

### Detection of caspase activity

Cleaved caspase-3 and caspase-9 activities were investigated by colorimetric assays using respective colorimetric kits according to the manufacturer’s protocol. Briefly, mouse chondrocytes treated by IL-1β or IL-1β + *L. wallichii* and untreated cells were washed twice with ice-cold PBS and lysed in lysis buffer for 10 min on ice. The cell lysates were centrifuged at 14,000×*g* for 10 min, and the resulting supernatants were employed to determine caspase activity by supplement of 5 μl caspase substrate and incubated in a 96-well plate for 4 h at 37 °C in a CO_2_ incubator. Finally, the absorbance was read at 405 nm in a microplate reader (Biotek). Relative caspase-3 or caspase-9 activity was calculated as a ratio of drug-treated cells to untreated cells.

### Flow cytometric analysis of apoptosis

For flow cytometry, a FITC-labeled recombinant Annexin V apoptosis detection kit (Beyotime) was employed. Treated or untreated cells were manipulated by unceasingly harvesting, washing in PBS and reconstituting in coupling buffer (10 mM HEPES/NaOH, pH 7.4, 140 mM NaCl, 2.5 mM CaCl2). Annexin V-FITC was added to a final concentration of 250 ng/ml prior to incubation in darkness at 4 °C for 15 min, then washed in PBS and re-suspended in 190 µl of coupling buffer, followed by 10 µl of propidium iodide (PI) for a further 5 min. Stained cells were analyzed using a FACStar plus flow cytometer (Becton–Dickinson). The ratio of fluorescence intensities excited at 488 nm was monitored at an emission wavelength of 515 nm for FITC and 560 nm for PI. Data analysis was performed with a BD BioSciences FACSCalibur flow cytometer using CellQuest software.

### Quantitative real-time PCR

The mouse chondrocytes were subjected to Trizol reagent to extract the total RNA following the manufacture’s instruction. After quantifying by NanoDrop spectrophotometer, the equal amount of total RNA was utilized to reverse transcription to cDNA using SuperScript^®^ III First-Strand Synthesis SuperMix kit (Invitrogen). The cDNA product was then amplified using Platinum^®^ SYBR Green qPCR SuperMix-UDG with Rox kit (Invitrogen) depending on the primers (PrimerBank). The copy numbers of each gene were calculated by cycle threshold (ΔCt) methods. Means of the copy numbers of GAPDH were employed as internal controls to normalize the data.

### Western blotting analysis

For immunodetection, mouse chondrocytes were lysed directly for 30 min in the Lysis Solution (0.5% SDS, 1% NP-40, 1% sodium deoxycholate, 150 mM NaCl, 50 mM pH 7.5 Tris–HCl, and protease inhibitors). Lysates were separated by centrifugation (13,000×*g*, 30 min, 4 °C) and 50 µg of total proteins was electrophoresed on a 10% or 12% SDS-PAGE, which was transferred onto polyvinylidene difluoride (PVDF) membranes in a transfer tank using transfer buffer (195 mM glycine, 25 mM Tris–HCl and 20% (v/v) methanol). The first stained membrane was confirmed the transfer efficiency with Ponceau S. Then the PVDF membranes were blocked for 1 h at RT with 3% (w/v) bovine serum albumin (BSA) in Tris-buffered saline (50 mM Tris, pH 8.0, 150 mM NaCl) with 0.05% Tween 20 (TBS-T). Membranes were incubated by the primary antibodies against p-p65, p65, p-κBα, κBα, MMP-13, Coll X, Aggrecan, Coll II, and GAPDH, then followed by secondary antibody conjugated with horseradish peroxidase at 1:2000 dilutions. Positive band intensities were shown by utilizing a gel documentation system (LAS-3000 Fujifilm).

### Derivatization and GC/MS analysis

Deriving mouse chondrocyte samples was required prior to GC/MS analysis. After drying samples, 80 μl of methoxamine/pyridine hydrochloride (20 mg/ml) was added to induce oximation for 1.5 h at 37 °C and then 80 μl of MSTFA, a derivatization reagent (Sigma), was mixed and reacted with the cell sample for additional 0.5 h at 37 °C. By centrifuging, 1 μl of supernatant derivative was added to a tube and analyzed using GC/MS (Trace DSQ II, Thermo Scientific). The separation conditions of GC/MS consisted of an initial temperature of 70 °C (5 min) with a uniform increase to 270 °C at a speed of 2 °C/min (5 min); 0.5 μl sample volume, splitless injection; injection temperature, 270 °C; interface temperature, 270 °C; ion source (EI) temperature, 30 °C; ionization voltage, 70 eV; quadrupole temperature, 150 °C; carrier gas, highly pure helium; velocity, 1.0 ml/min; and full scan way, 60–600 m/z.

### Statistical and bioinformatics analysis

The data of mouse chondrocyte metabolome were collected using Thermo Foundation 1.0.1. The sum abundance value was employed for normalizing the resulting data matrix, and then the computed abundance of metabolites was centered for each tissue sample on their median value and scaled by their inter-quartile range (IQR) to decline between-sample variation [17, 18]. The significant analysis of microarray (SAM), a permutation-based hypothesis testing method for the analysis of metabolomic data [19, 20], was applied to analyze the differential metabolites. Principal component analysis (PCA) was chosen as the pattern recognition method [21]. Orthogonal partial least square discriminant analysis (OPLS-DA) with software SIMCA 12.0 (Umetrics, Umeå, Sweden) was used to identify patterns associated with IL-1β treatment or IL-1β + *L. wallichii* treatment and minimize influence of the interindividual variation. Individuals with different phenotypes in the same group were termed interindividual variation [17]. Statistical significance between groups was determined with the unpaired two-tailed Student *t* test. All data were analyzed by Prism (GraphPad Software, Inc.), and *p* values less than 0.05 and 0.01 were deemed as two significant levels.

## Results

### *Ligusticum wallichii* extracts protect mouse chondrocytes against IL-1β-induced apoptosis

**Fig. 1:**
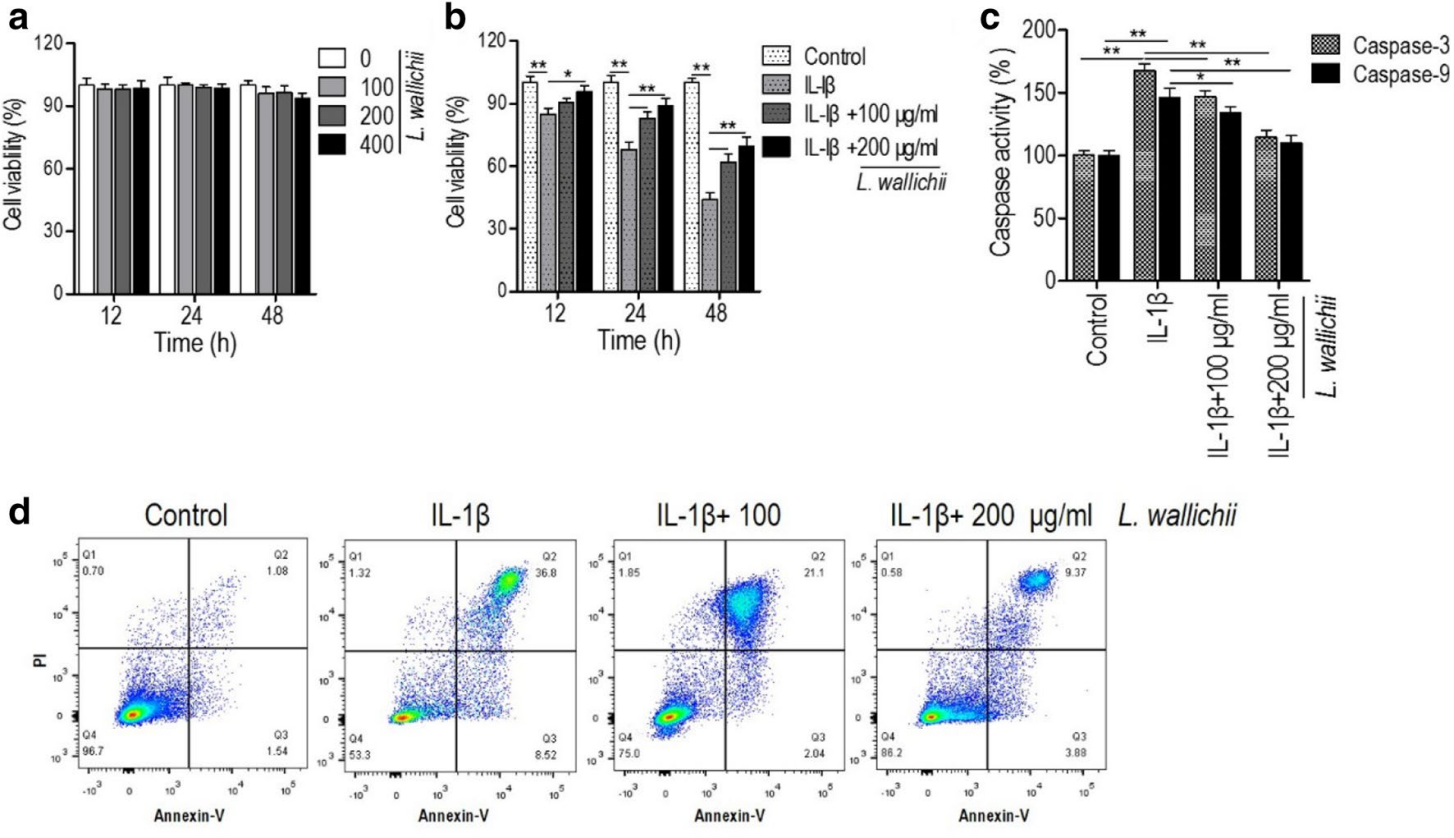
*Ligusticum wallichii* extracts increased the cell viability and decreased the apoptosis of IL-1β-stimulated primary mouse chondrocytes. **a** *L. wallichii* alone had no significant impact on the viability of mouse chondrocytes. **b** IL-1β reduced the cell viability of primary mouse chondrocyte, which was rescued by *L. wallichii* treatment. Two concentrations (100 and 200 μg/ml) of *L. wallichii* extracts were used. The control was treated by the same volume of DMSO. **c** *L. wallichii* extracts reduced the caspase-3 and caspase-9 activities on IL-1β-stimulated cells. The treatment condition was same as **b**. **d** Flow cytometric analysis of the apoptosis of primary mouse chondrocytes treated by IL-1β or IL-1β +* L. wallichii*. Error bars ± SEM, *p < 0.05, **p < 0.01

As shown in Fig. 1a, only *L. wallichii* treatment (from 100 to 400 μg/ml) had no obvious impact on the viability of primary mouse chondrocytes. IL-1β is the main contributor to OA pathology and commonly used as an inducer in in vitro inflammatory model of chondrocyte [22]. In line with previous report [3, 23], IL-1β treatment significantly decreased the cell viability of mouse chondrocyte in a time-dependent manner (Fig. 1b). The incubation of *L. wallichii* extract initiated the alleviation of IL-1β-induced damage at low concentration (100 μg/ml) and further enhanced the cell viability of IL-1β-injured chondrocyte at high concentration (200 μg/ml) (Fig. 1b). Then we addressed whether the effect of *L. wallichii* extract on IL-1β-treated chondrocyte resulted from the inhibition of cell apoptosis. Firstly, we determined the activities of caspase-3 and caspase-9 in mouse chondrocytes treated either with IL-1β or IL-1β plus *L. wallichii* extract. As shown in Fig. 1b, IL-1β treatment significantly enhanced the caspase-3 and caspase-9 protease activities. As expected, *L. wallichii* extracts suppressed both caspase activities in a dose-dependent manner (Fig. 1c). Employing the promotion of Annexin V^+^/PI^−^ (Annexin V^+^/PI^+^ represents the cell necrosis) fluorescence intensity as readout for increased apoptosis, we discovered that mouse chondrocyte treated with IL-1β showed significant induction in cell apoptosis in contrast to untreated control and the usage of *L. wallichii* extract rescues the IL-1β-induced apoptosis in a dose-dependent manner (Fig. 1d). Collectively, these data indicate that *L. wallichii* extract is an efficient drug that inhibits IL-1β-induced injury of mouse chondrocyte through reducing the apoptosis.

### *Ligusticum wallichii* extracts relieve inflammatory response and extracellular matrix (ECM) degradation in IL-1β-injured mouse chondrocytes

**Fig. 2:**
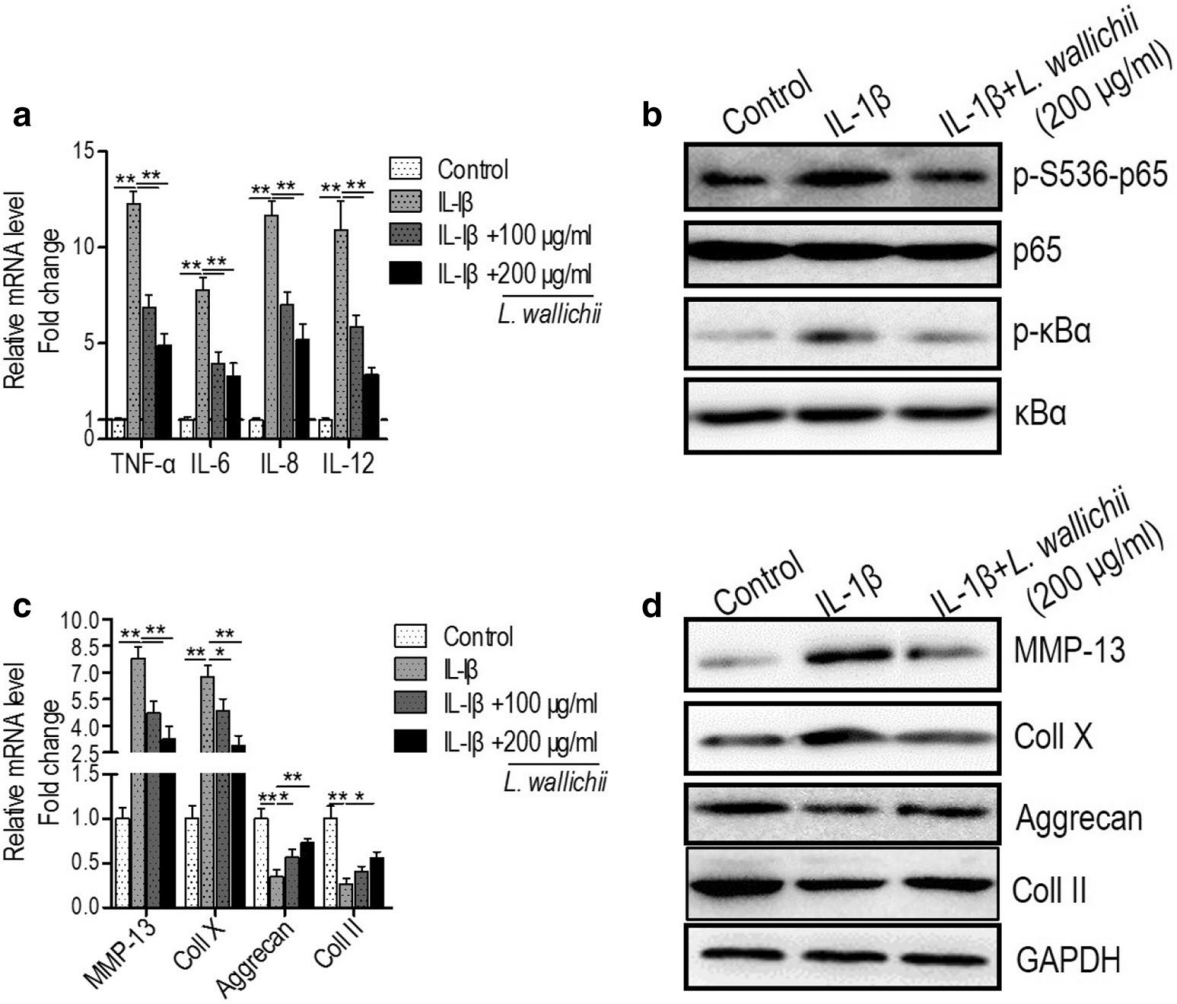
*L. wallichii* extracts alleviate inflammatory response and extracellular matrix (ECM) degradation in IL-1β-administrated primary mouse chondrocytes. **a** The relative mRNA levels of TNF-α, IL-6, IL-8, and IL-12 were detected in primary mouse chondrocytes treated by IL-1β or IL-1β +* L. wallichii*. **b** Western blotting was used to investigate the cellular level of p-p65, p65, p-κBα, and κBα protein. **c** The relative mRNA levels of MMP-13, Coll X, Aggrecan, and Coll II were detected in primary mouse chondrocytes treated by IL-1β or IL-1β +* L. wallichii*. **d** Western blotting was used to investigate the cellular level of MMP-13, Coll X, Aggrecan, and Coll II. Error bars ± SEM, *p < 0.05, **p < 0.01

To investigate the effect of *L. wallichii* extract on the inflammatory response in IL-1β-treated mouse chondrocytes, the transcriptional levels of inflammatory cytokines TNF-α, IL-6, IL-8, and IL-12 were estimated and the results showed that IL-1β-induced mRNA levels of TNF-α, IL-6, IL-8, and IL-12 were obviously reduced by co-incubation with *L. wallichii* extract (Fig. 2a). Furthermore, the NF-κB signaling that activated by IL-1β was repressed by *L. wallichii* extract suggesting by the down-regulation of p-p65 and p-κBα (Fig. 2b). The dyshomeostasis of ECM degradation and synthesis of chondrocytes is one of the critical reasons for the degeneration of cartilage ^23^. To check the effect of *L. wallichii* extract on the ECM degradation, the expressions, and productions of the enzyme that catalyzes the degradation of ECM and ECM components were identified. The outcomes presented that the transcriptional and translational levels of matrix metalloproteinase 13 (MMP-13) and Coll X were dramatically induced and the mRNA and protein levels of Coll II and Aggrecan were markedly boosted in IL-1β-treated chondrocytes (Fig. 2c, d). Interestingly, all the above-mentioned changes in terms of ECM degradation were reserved by the usage of *L. wallichii* extract (Fig. 2c, d). Altogether, the above data suggest that *L. wallichii* extract alleviates IL-1β-triggered inflammatory reaction and ECM degradation in mouse chondrocyte.

### GC/MS dissects the metabolomics profiling of mouse chondrocyte

**Fig. 3:**
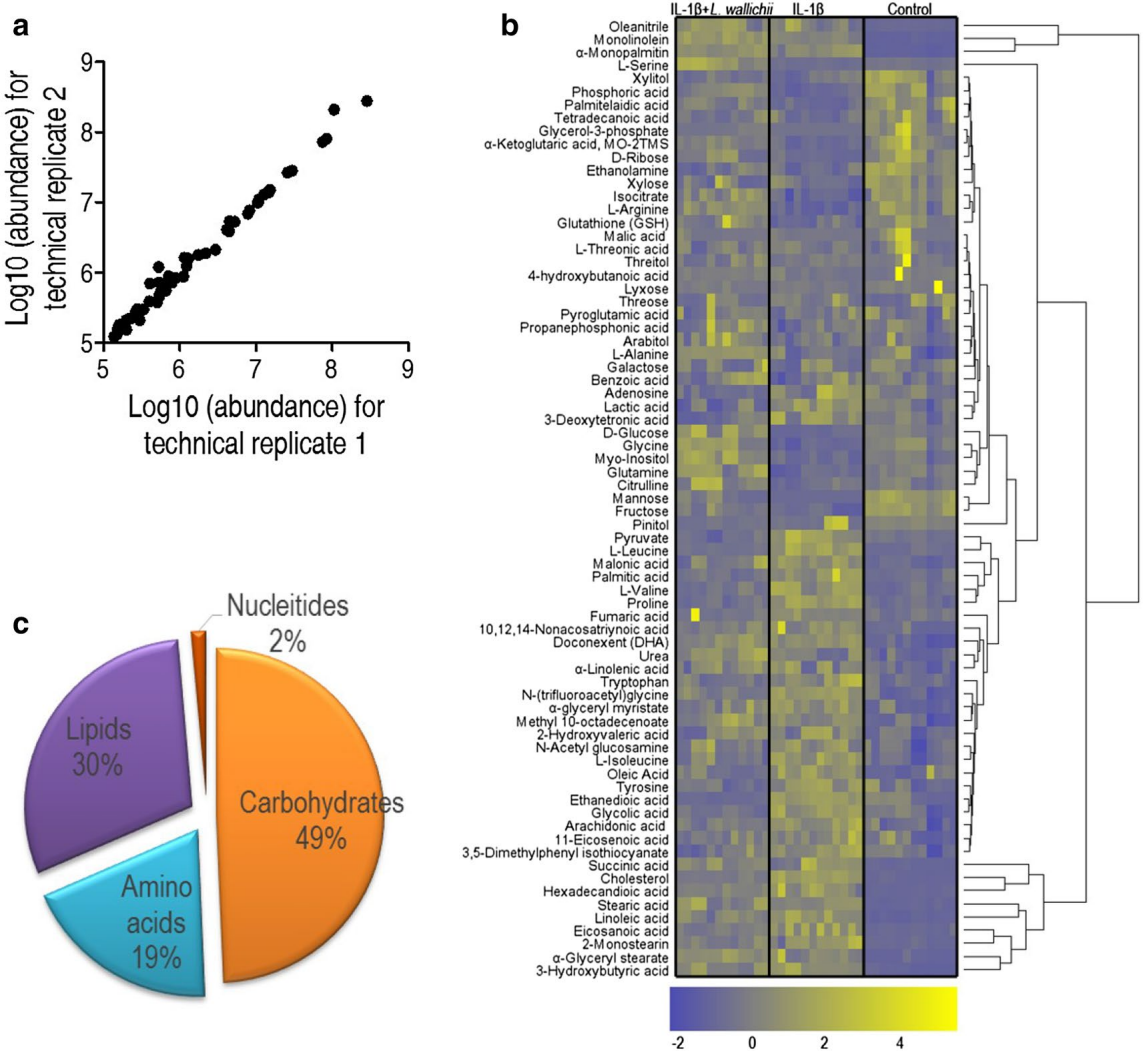
Metabolomics profiling of primary mouse chondrocyte treated by IL-1β or IL-1β +* L. wallichii*. **a** Reproducibility of metabolomic data used in the discovery phase. Metabolite abundances quantified in cell samples over two technical replicates are shown. Correlation coefficient between technical replicates varies between 0.995 and 0.999. This plot presents the two replicates with the weakest correlation of 0.995. **b** Hierarchical clustering showing the 73 metabolites. Blue and yellow present decrease and increase of metabolites relative to the median metabolite level, respectively (see color scale). **c** Metabolic category of 73 identified metabolites

To exploit the critical metabolic pathways and key metabolites that are potentially responsible for the beneficial outcomes of *L. wallichii* extract on IL-1β-injured chondrocyte, GC/MS was employed to quantitatively measure the level of known metabolites in mouse chondrocytes. The correlation coefficient of two technical repeats represented the reliability of the detection technology (Fig. 3a). As shown in Fig. 3b, 73 metabolites with reliable signals were identified in each sample and clustered as a heat map. Biological roles of metabolites were defined according to KEGG. The category showed that 49%, 19%, 30% and 2% of metabolites classified into carbon sources, amino acids, lipids, and nucleotides, respectively (Fig. 3c), suggesting the establishment of carbohydrates-, amino acids- and lipids-dominant metabolome of mouse chondrocytes.

### Differential metabolome associated with IL-1β and *L. wallichii* treatment

**Fig. 4:**
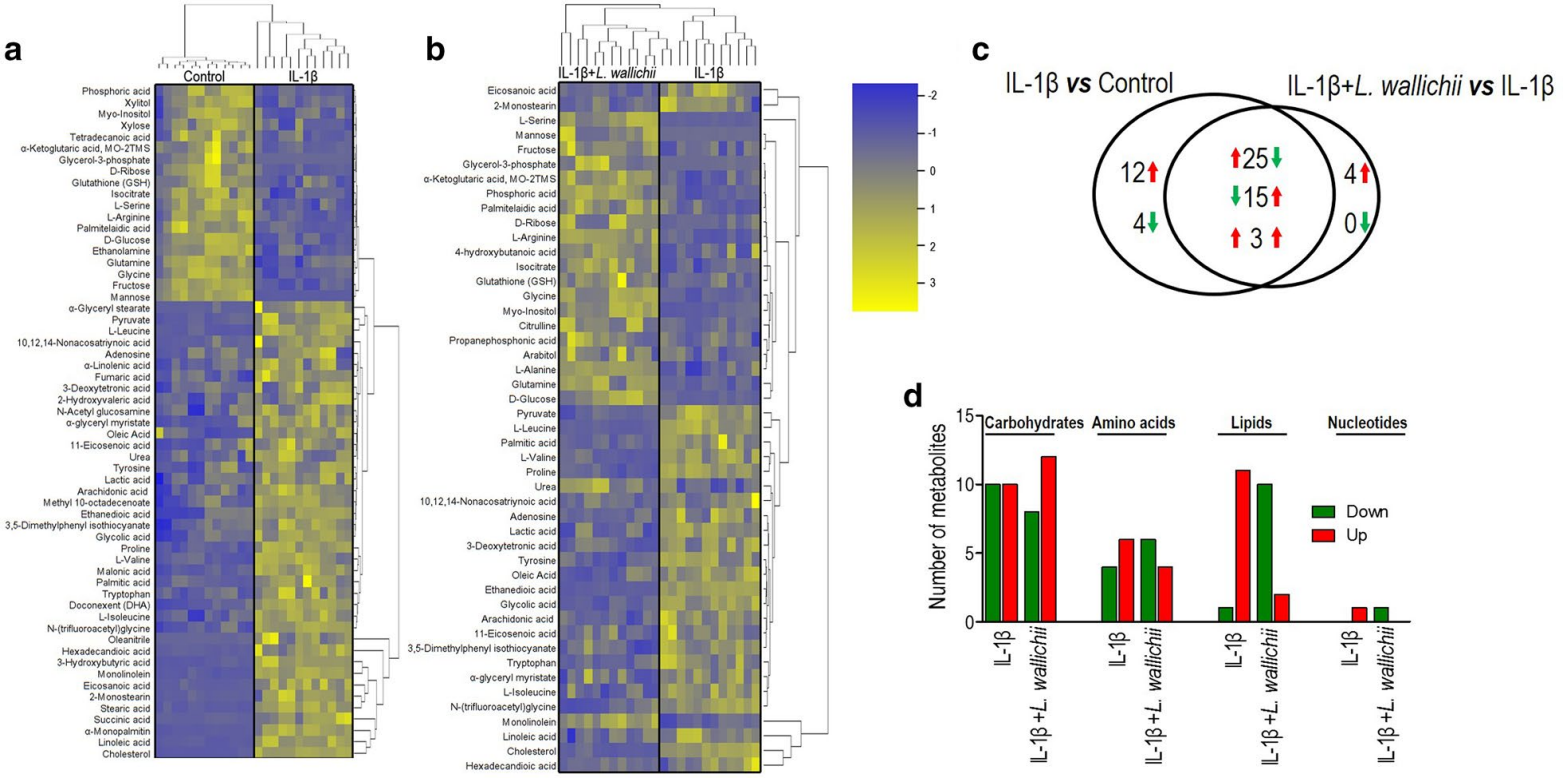
Differential metabolome related to IL-1β and *L. wallichii* treatment. **a** Heat map for relative abundances of differential metabolites (*n* = 59) in IL-1β-treated primary mouse chondrocytes compared with the untreated cells. **b** Heat map for relative abundances of differential metabolites (*n* = 47) in IL-1β +* L. wallichii*-treated primary mouse chondrocytes compared with IL-1β-treated primary mouse chondrocytes. Blue and yellow present decrease and increase of metabolites relative to the median metabolite level, respectively (see color scale). **c** Venn diagram exhibiting the overlap of differential metabolites between the IL-1β and IL-1β + *L. wallichii* groups. Up-regulated and down-regulated metabolites are indicated with red and green arrows, respectively. **d** The number of metabolites increased and decreased in different metabolic categories

To further assess a varied metabolome identifying IL-1β + *L. wallichii*-treated group from the IL-1β-treated group, a two-sided Wilcoxon rank-sum test coupled with a permutation test was used to explore differential metabolites. Fifty-nine (80.82%) and forty-seven (64.38%) metabolites with differential abundances were respectively obtained from the comparison of Control and IL-1β groups and of IL-1β and IL-1β + *L. wallichii* groups, among which 43 metabolites were shared (Fig. 4a–c). Hierarchical clustering was used to arrange the metabolites on the basis of their relative levels across samples (Fig. 4a, b). Specifically, 40 metabolites were up-regulated and 19 metabolites were down-regulated in IL-1β-treated group, while 22 metabolites were up-regulated and 25 metabolites were down-regulated in IL-1β + *L. wallichii*-treated group (Fig. 4a, c). In addition to the shared metabolites, twelve metabolites were increased, and four metabolites were decreased in IL-1β group, whereas only four metabolites were increased in IL-1β + *L. wallichii* group (Fig. 4c). Among the 43 shared metabolites, three metabolites were simultaneously increased in IL-1β and IL-1β + *L. wallichii* groups, 25 metabolites were increased in IL-1β group but decreased in IL-1β + *L. wallichii* group and 15 metabolites were decreased in IL-1β group but increased in IL-1β + *L. wallichii* group (Fig. 4c), indicating that the abundance of most of the metabolites (*n *= 40) altered by IL-1β stimulation could be repressed by *L. wallichii* treatment. Metabolic categories of these differential metabolites in abundance were estimated further. Lipid metabolism was likely to be more affected by *L. wallichii* treatment (Fig. 4d). These data reveal that a change in metabolites is related to a beneficial outcome triggered by *L. wallichii* extract.

### Identification of crucial metabolites using multivariate data analysis

**Fig. 5:**
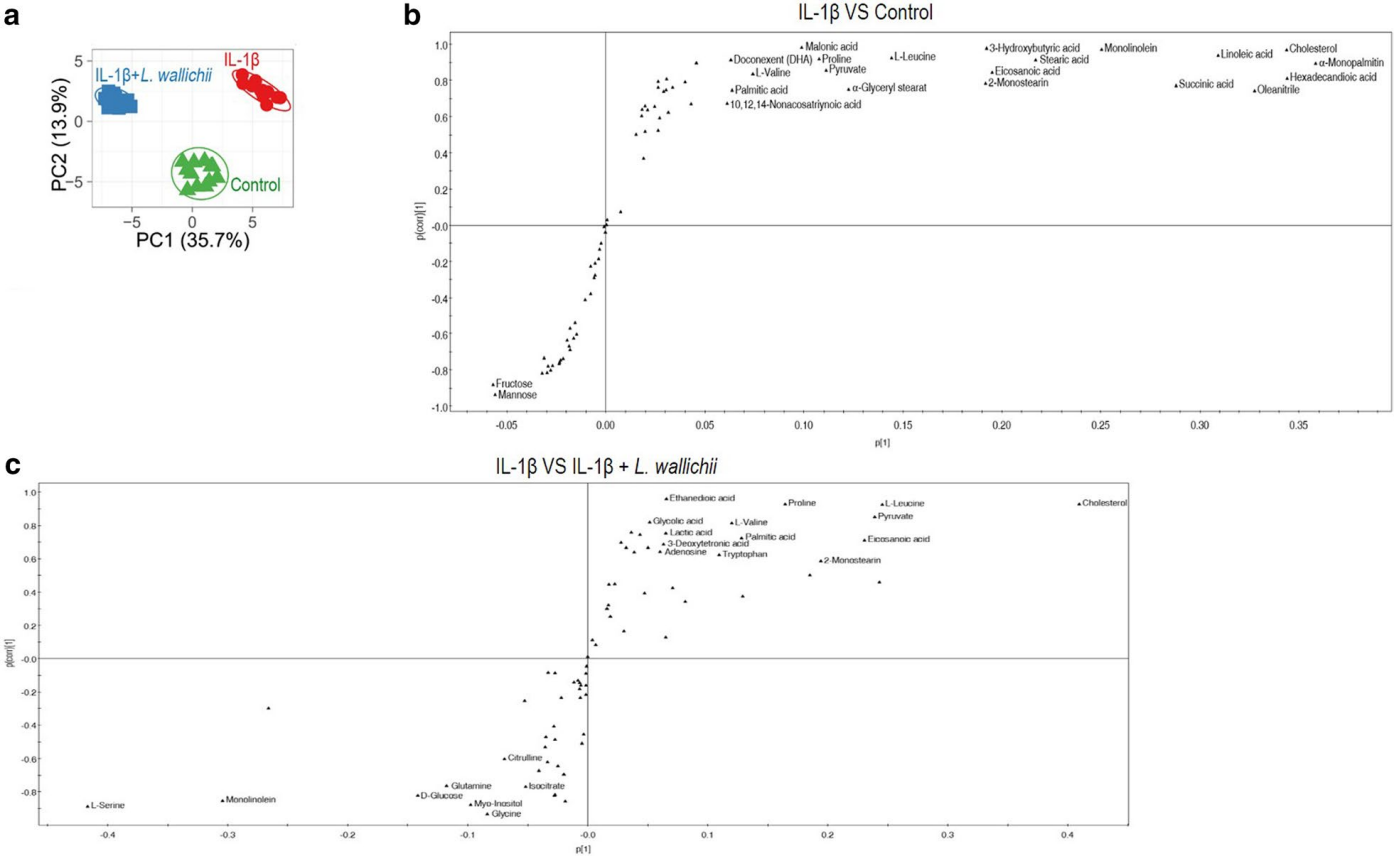
Multivariate data analysis. Pathway **a** PCA directly suggests a variation of metabolites among control, IL-1β-treated, and IL-1β +* L. wallichii*-treated group. Each dot in the panel indicates the replicate analysis of samples. **b**, **c** S-plot generates from OPLS-DA. Predictive component p [1] and correlation p(corr) [1] explain the difference between control and IL-1β, and IL-1β and IL-1β + *L. wallichii*

To explore the most crucial metabolites causing the beneficial effect of *L. wallichii* in IL-1β-treated mouse chondrocytes, OPLS-DA was performed to recognize the sample pattern. The distribution was shown in Fig. 5a, indicating an interesting correlation between the metabolomics responses and degree of IL-1β-induced injury. First component and second component explained 35.7% and 13.9% of *R*^2^(X) variations in the metabolic pattern, respectively. Discriminating variables were presented by S-plot (Fig. 5b, c). Cut-off values were set as greater or equal to 0.05 and 0.5 for absolute value of covariance p and correlation p(corr), respectively [17]. Twenty-one metabolites contributing to discriminate IL-Iβ treatment group from control group were shown in Fig. 5b. It contained 19 positive correlation metabolites (α-Monopalmitin, cholesterol, hexadecandioic acid, oleanitrile, linoleic acid, succinic acid, monolinole, streatic acid, eicosanoic acid, 2-monostearin, 3-hydroxybutyric acid, l-leucine, α-glyceryl myristate, pyruvate, proline, malonic acid, l-valine, palmitic acid, and DHA) and only two negative correlation metabolites (fructose and mannose). Twenty-four metabolites could be employed as potential targets for *L. wallichii* treatment (Fig. 5c). Out of them, 16 with negative correlation were cholesterol, hexadecandioic acid, l-leucine, pyruvate, eicosanoic acid, 2-monostearin, linoleic acid, proline, l-valine, palmitic acid, ethanedioic acid, glycolic acid, lactic acid, 3-deoxytetronic acid, adenosine, and tryptophan, and eight with positive correlation were l-serine, monolinole, d-glucose, glutamine, myo-inositol, glycine, citrulline, isocitrate. When compared the readouts between Fig. 5b, c, cholesterol, linoleic acid, hexadecandioic acid, proline, l-valine, l-leucine, pyruvate, palmitic acid, and proline are the most key biomarkers that specifically related to both IL-1β-mediated injury and *L. wallichii*-mediated protection since they were appeared on both IL-1β and IL-1β + *L. wallichii* treatments.

### Characterization of metabolic pathways involved in beneficial response induced by *L. wallichii* extracts

**Fig. 6:**
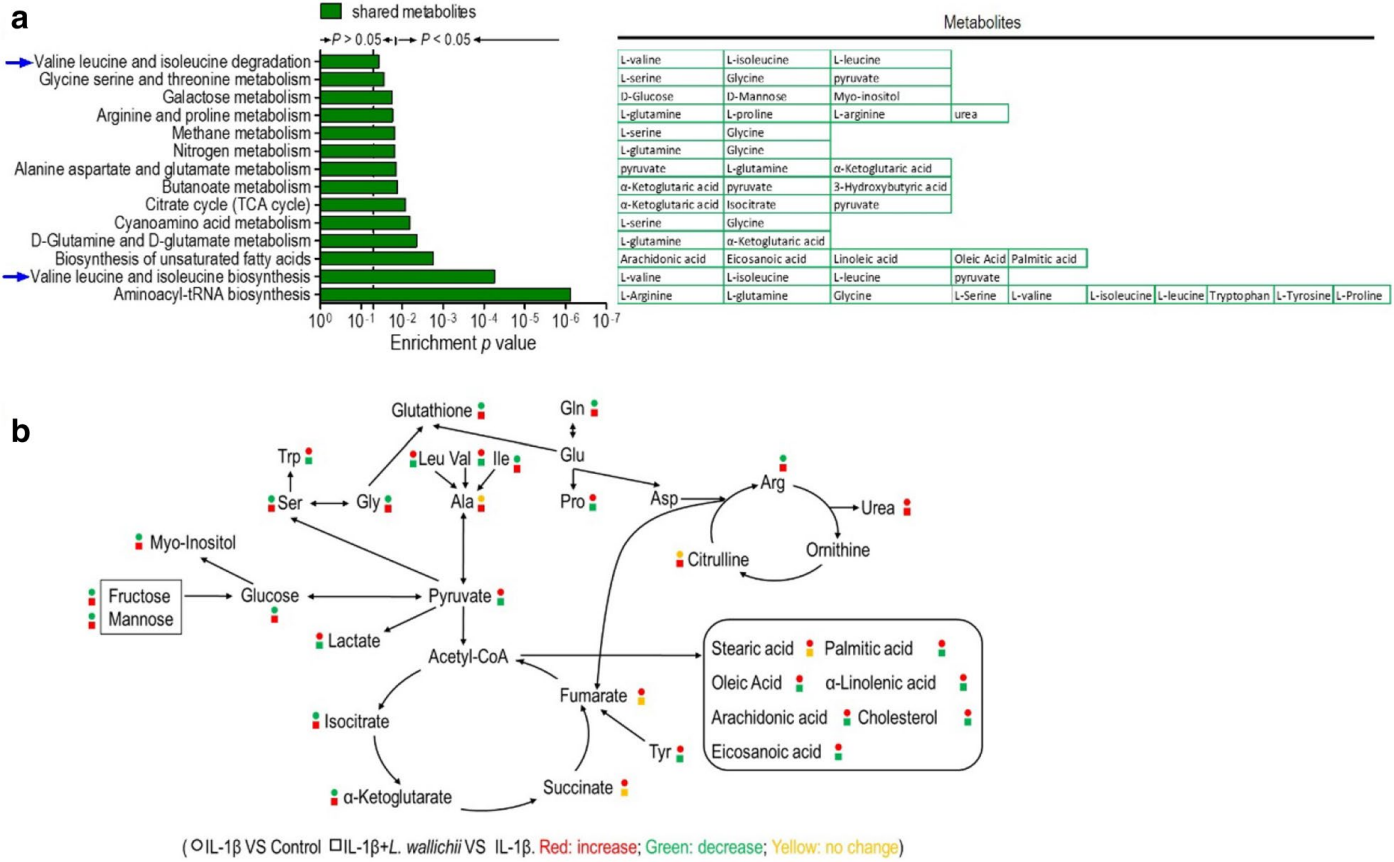
Pathway enrichment analysis and potential metabolic mechanisms of IL-1β-induced injury in chondrocytes and protection by *L. wallichii*. **a** By using an online tool, Metaboanalyst 4.0 (http://www.metaboanalyst.ca/), several pathways were enriched. The corresponding metabolites were shown in every metabolic pathway. **b** The metabolic flow of primary mouse chondrocytes treated by IL-1β or IL-1β + *L. wallichii*. Symbol circle and square represent the relative metabolite changes in the IL-1β group and the IL-1β + *L. wallichii* groups, respectively. The decrease, increase and no change in levels with statistical significance are presented in green, red and yellow, respectively

All shared differential metabolites shown in Fig. 4c were used to enrich the metabolic pathways by Metaboanalyst 4.0. Totally fourteen pathways were acquired when using *p* < 0.05 as an evaluation parameter (Fig. 6a). Among them, eight pathways, aminoacyl-tRNA biosynthesis, valine, leucine and isoleucine biosynthesis, d-glutamine and d-glutamate metabolism, cyanoamino acid metabolism, alanine, aspartate, and glutamate metabolism, arginine and proline metabolism, glycine, serine, and threonine metabolism, and valine, leucine and isoleucine metabolism, are related to amino acid metabolisms. Furthermore, four pathways containing citrate cycle, butanoate metabolism, methane metabolism, and galactose metabolism are involved in carbohydrate metabolisms, and only one pathway called biosynthesis of unsaturated fatty acids is one of the lipid metabolisms. Also, all pathway-involved metabolites were described in Fig. 6a and further visualized in Fig. 6b for understanding the metabolic flow of mouse chondrocytes by IL-1β or IL-1β + *L. wallichii* treatment.

## Discussion

*Ligusticum wallichii* is usually recognized as a drug that protects host cells against inflammation since the major effective components including ferulic acid, ligustrazine, and tetramethylpyrazine extracted from this herb are able to alleviate the inflammation-induced injury in different cells or tissues [13, 24–26]. However, whether *L. wallichii* may have a protective role in chondrocytes upon inflammatory condition remains sealed. The present study suggests that *L. wallichii* extracts function as a protective agent in mouse chondrocytes injured by IL-1β, a major pathological contributor to OA [22, 27], via attenuating the IL-1β-induced apoptosis, inflammatory response, and extracellular matrix (ECM) degradation. Recent demonstrations indicate that metabolic disorder is responsible for the pathogenesis of OA [2]. To date, IL-1β is known in reducing mitochondrial oxidative phosphorylation (MOP) by switching to glycolysis and producing excessive reactive oxygen species (ROS) in chondrocytes through damaging mitochondrial structure, dynamics, and genome stability, resulting in mitochondrial dysfunction and oxidative stress, which are two main hallmarks of OA [2, 28]. Our metabolomics analysis also evidences the accelerated glycolysis and dysfunctional MOP as excessive consumption of glucose, fructose, and mannose, an excess of pyruvate and lactate, reversed level of four intermediates (fumarate, succinate, α-ketoglutarate, and isocitrate) in TCA cycle are identified. However, besides the MOP, whether IL-1β acts as a mediator influencing other metabolic pathways is undiscovered so far. Also, it is unknown whether *L. wallichii* extracts are capable of mounting metabolic strategies to restore the IL-1β-triggered injury. Therefore, in follow-up studies, we continually focus on the investigation of metabolic response of IL-1β-treated and IL-1β + *L. wallichii*-treated chondrocytes through employing GC/MS-based metabolomics. Our study not only reveals the reprogramming of glycolysis and TCA cycle upon *L. wallichii* treatment but also clues that the alteration of other metabolic responses is potentially related to the protective effect of *L. wallichii* in IL-1β-injured chondrocytes due to the exploration of valuable pathways and metabolites.

Currently, the metabolic category shows that *L. wallichii* extracts decline the lipid metabolism, the following pathway enrichment analysis further clarifies that biosynthesis of unsaturated fatty acids probably involves in the *L. wallichii*-triggered metabolic response. This metabolism represents five metabolites, arachidonic acid, eicosanoic acid, linoleic acid, oleic acid, and palmitic acid, which are all increased by IL-1β treatment but decreased by IL-1β + *L. wallichii* treatment. Another fatty acid up-regulated by IL-1β here is stearic acid. Actually, earlier report shows that levels of several fatty acids, palmitic acid, stearic acid, oleic acid, linoleic acid, and arachidonic acid, were markedly boosted in association with increasing level of lesion severity in human osteoarthritic articular cartilage [29]. Moreover, OA induced by destabilizing the medial meniscus in mice is significantly associated with dietary fatty acid content [30]. Particularly, diets enriching saturated fatty acids (etc., palmitic acid and stearic acid) or ω-6 polyunsaturated fatty acids (etc., arachidonic acid and linoleic acid) independently aggravate OA severity. Palmitic acid serves as a proinflammatory and catabolic factor, in synergy with IL-1β, triggers chondrocyte apoptosis and articular cartilage deterioration through Toll-like receptor 4 pathway [31]. In primary mouse chondrocytes, stearic acid administration leads to the stabilization of hypoxia-inducible factors 1α (HIF1α), a marker of oxidative stress, and the increase of several proinflammatory cytokines including IL-6, IL-1β, and TNF-α [32]. Thus, blocking the synthesis of pro-inflammatory fatty acids mentioned above is a potential metabolic mechanism for *L. wallichii*-induced protection against IL-1β injury in chondrocytes.

Besides the ROS, nitric oxide (NO) also plays a significant role in mediating oxidative stress, a major hallmark of OA. The role of NO production in OA pathogenesis has been exclusively investigated [33]. l-Arginine is the exclusive resource of NO in mammalian cells and can be additionally metabolized by arginase. The latest study indicates that arginase II is also an important regulator of OA pathogenesis in mice at least partly by upregulating the expression of MMP-13 in chondrocytes via the NF-κB signaling [34]. Upon IL-1β stimulation, chondrocytes have upregulated expression of arginase II and inducible nitric oxide synthase (iNOS) [34], indicating the high demand and consumption of l-arginine in OA chondrocytes. Interestingly, metabolomics analysis of human plasma explores that l-arginine is the most differential metabolite with knee OA patients owning on average 69 μM lower than that in healthy controls [35], which in line with our outcome that IL-1β treatment decreases the abundance of l-arginine and increases the level of urea (the enzymatic product of arginase II) in chondrocytes. On the other hand, oxidative stress can be alleviated by one body’s antioxidant, glutathione, the latter is a tri-peptide (glutamate–cysteine–glycine) that serves to neutralize peroxide free radicals [36]. All three amino acid components are directly and indirectly managed by l-glutamine. First of all, glutaminase converts l-glutamine into l-glutamate in one step, l-glutamate serves as a nitrogen donor for the transamination that generates the l-alanine, l-aspartate, and l-serine. Then serine hydroxymethyltransferase accomplishes the subsequent conversion of serine to glycine, and glutamate can be exchanged for cysteine through the xCT antiporter. This antiporter is the rate-limiting factor for the glutathione synthesis and significantly reduced in OA rats [37]. In the present study, IL-1β-induced reduction of abundance of l-serine, glycine, l-glutamine, and glutathione are found to be boosted by *L. wallichii* treatment, revealing an interesting potential that modulation of the level of these metabolites can partly explain how *L. wallichii* imparts strong protection in IL-1β-injured chondrocytes.

We found two remarkable amino acid metabolisms in pathway analysis, which are l-valine, l-leucine, and isoleucine biosynthesis and degradation. Given that l-valine, l-leucine, and isoleucine are three essential amino acids in mammalian, the stronger abundance of l-valine, l-leucine, and l-isoleucine indicates the inhibition of l-valine, l-leucine, and l-isoleucine degradation in IL-1β-administrated chondrocytes. Several metabolomics studies intensively demonstrate enhanced levels of l-valine, l-leucine, and l-isoleucine in OA patients compared to levels in healthy control [38–40]. The upregulated concentration of l-valine, l-leucine, and l-isoleucine can results in a rising production of cytokines, thereby leading to an enhanced degree of joint collagen degradation [41]. Moreover, previous study reports that l-leucine is capable of inducing high activity of bone morphogenetic protein 2 (BMP2) and the high-level BMP2 is strongly related to the severe aggravation of osteophyte formation [42, 43]. One pathway upstream of BMP2, mechanistic target of rapamycin (mTOR), can be respectively regulated by l-valine, l-leucine, and l-isoleucine [44, 45]. Aberrant mTOR signaling associated with peroxisome proliferator-activated receptor γ deficiency leads to severe and accelerated OA [46]. More interesting, l-valine, l-leucine, and l-isoleucine have a function in upregulating the NO production by suppressing the activity of Arginase [18, 47, 48]. Thus improving the degradation of l-valine, l-leucine, and l-isoleucine is an underlying metabolic mechanism for *L. wallichii*-induced protection in IL-1β-injured chondrocyte.

## Conclusions

The current study explores that *L. wallichii* reduces the IL-1β-induced apoptosis, inflammatory response, and extracellular matrix (ECM) degradation in mouse chondrocytes. The GC/MS-based metabolomics analysis shows that the abundance of most of the metabolites (*n *= 40) altered by IL-1β stimulation could be repressed by *L. wallichii* treatment. Further pathway analysis using these metabolites enriched fourteen metabolic pathways, which were dramatically changed in IL-1β-treated chondrocytes and capable of being reprogrammed by *L. wallichii* incubation. These enriched pathways were involved in carbon metabolisms, fatty acid biosynthesis, and amino acid metabolisms. Altogether, these findings offer potential clues that metabolic strategies are linked to protective mechanisms of *L. wallichii* treatment in IL-1β-stimulated chondrocytes and emphasize the importance of metabolic strategies against inflammatory responses in OA development.

## Data Availability

All original data supported the discovery of current study were supplied by Xinzhu Wen under license and cannot be made freely available. Requests for access to these data should be made to Xinzhu Wen, wenxinzhu_hospi1@163.com.

## Abbreviations

OA: osteoarthritis
ECM: extracellular matrix
PI: propidium iodide
PVDF: polyvinylidene difluoride
BSA: bovine serum albumin
EI: ion source
IQR: their inter-quartile range
SAM: significant analysis of microarray
PCA: principal component analysis
OPLS-DA: orthogonal partial least square discriminant analysis
MMP-13: matrix metalloproteinase 13
MOP: mitochondrial oxidative phosphorylation
ROS: reactive oxygen species
NO: nitric oxide
BMP2: bone morphogenetic protein 2
mTOR: mechanistic target of rapamycin

## References

[1] Berenbaum F (2013). Osteoarthritis as an inflammatory disease (osteoarthritis is not osteoarthrosis!). Osteoarthritis Cartilage..

[2] Mobasheri A, Rayman MP, Gualillo O, Sellam J, Van Der Kraan P, Fearon U (2017). The role of metabolism in the pathogenesis of osteoarthritis. Nat Rev Rheumatol.

[3] Bai M, Ge L, Chen H, Jin Q (2019). Calcitonin protects rat chondrocytes from IL-1β injury via the Wnt/β-catenin pathway. Exp Ther Med.

[4] Glyn-Jones S, Palmer A, Agricola R (2015). Osteoarthritis. Lancet..

[5] Gomez R, Lago F, Gomez-Reino J, Dieguez C, Gualillo O (2009). Adipokines in the skeleton: influence on cartilage function and joint degenerative diseases. J Mol Endocrinol.

[6] Michalek RD, Rathmell JC (2010). The metabolic life and times of a T-cell. Immunol Rev.

[7] van der Kraan P, Matta C, Mobasheri A (2017). Age-related alterations in signaling pathways in articular chondrocytes: implications for the pathogenesis and progression of osteoarthritis-a mini-review. Gerontology..

[8] Mobasheri A, Matta C, Zákány R, Musumeci G (2015). Chondrosenescence: definition, hallmarks and potential role in the pathogenesis of osteoarthritis. Maturitas..

[9] Sellam J, Berenbaum F (2013). Is osteoarthritis a metabolic disease?. Joint Bone Spine..

[10] Kluzek S, Newton J, Arden N (2015). Is osteoarthritis a metabolic disorder?. Br Med Bull.

[11] June RK, Liu-Bryan R, Long F, Griffin TM (2016). Emerging role of metabolic signaling in synovial joint remodeling and osteoarthritis. J Orthop Res.

[12] Hansen P (1995). Myocardial reperfusion injury: experimental evidence and clinical relevance. Eur Heart J.

[13] Jiang C, Peng Y, Fang X (2018). Ligusticum wallichii elevates the proliferation of hypoxia-stimulated human microglia through promoting HIF-1α-mediated MET expression. Int J Clin Exp Med..

[14] Tan F, Fu W, Cheng N, Meng D, Gu Y (2015). Ligustrazine reduces blood-brain barrier permeability in a rat model of focal cerebral ischemia and reperfusion. Exp Ther Med.

[15] Zhao Y, Liu Y, Chen K (2016). Mechanisms and clinical application of tetramethylpyrazine (an interesting natural compound isolated from *Ligusticum wallichii*): current status and perspective. Oxid Med Cell Long.

[16] Xie J, Fu N, Cai LY (2015). The effects of interleukin-1β in modulating osteoclast-conditioned medium’s influence on gelatinases in chondrocytes through mitogen-activated protein kinases. Int J Oral Sci.

[17] Guo C, Huang XY, Yang MJ (2014). GC/MS-based metabolomics approach to identify biomarkers differentiating survivals from death in crucian carps infected by Edwardsiella tarda. Fish Shellfish Immunol.

[18] Chen XH, Liu SR, Peng B (2017). Exogenous l-valine promotes phagocytosis to kill multidrug-resistant bacterial pathogens. Front Immunol.

[19] Chen XH, Zhang BW, Li H, Peng X-X (2015). Myo-inositol improves the host’s ability to eliminate balofloxacin-resistant *Escherichia coli*. Sci Rep.

[20] Su YB, Peng B, Li H (2018). Pyruvate cycle increases aminoglycoside efficacy and provides respiratory energy in bacteria. Proc Natl Acad Sci.

[21] Metsalu T, Vilo J (2015). ClustVis: a web tool for visualizing clustering of multivariate data using principal component analysis and heatmap. Nucleic Acids Res.

[22] Kapoor M, Martel-Pelletier J, Lajeunesse D, Pelletier JP, Fahmi H (2011). Role of proinflammatory cytokines in the pathophysiology of osteoarthritis. Nat Rev Rheumatol.

[23] Liu Q, Li M, Jiang L, Jiang R, Fu B (2019). METTL3 promotes experimental osteoarthritis development by regulating inflammatory response and apoptosis in chondrocyte. Biochem Biophys Res Commun.

[24] Yin P, Zhang Z, Li J (2019). Ferulic acid inhibits bovine endometrial epithelial cells against LPS-induced inflammation via suppressing NK-κB and MAPK pathway. Res Vet Sci.

[25] Hu JZ, Huang JH, Xiao ZM, Li JH, Li XM, Lu H-B (2013). Tetramethylpyrazine accelerates the function recovery of traumatic spinal cord in rat model by attenuating inflammation. J Neurol Sci.

[26] Li Y, Zhu Z, Zhang T, Zhou Y (2019). Ligustrazine attenuates inflammation and oxidative stress in a rat model of arthritis via the Sirt1/NF-κB and Nrf-2/HO-1 pathways. Arch Pharmacal Res.

[27] Page-McCaw A, Ewald AJ, Werb Z (2007). Matrix metalloproteinases and the regulation of tissue remodelling. Nat Rev Mol Cell Biol.

[28] Kim J, Xu M, Xo R (2010). Mitochondrial DNA damage is involved in apoptosis caused by pro-inflammatory cytokines in human OA chondrocytes. Osteoarthritis Cartilage..

[29] Lippiello L, Walsh T, Fienhold M (1991). The association of lipid abnormalities with tissue pathology in human osteoarthritic articular cartilage. Metabolism..

[30] Wu C-L, Jain D, McNeill JN (2015). Dietary fatty acid content regulates wound repair and the pathogenesis of osteoarthritis following joint injury. Ann Rheum Dis.

[31] Alvarez-Garcia O, Rogers NH, Smith RG, Lotz MK (2014). Palmitate has proapoptotic and proinflammatory effects on articular cartilage and synergizes with interleukin-1. Arthritis Rheumatol..

[32] Miao H, Chen L, Hao L (2015). Stearic acid induces proinflammatory cytokine production partly through activation of lactate-HIF1α pathway in chondrocytes. Sci Rep.

[33] Abramson SB (2008). Osteoarthritis and nitric oxide. Osteoarthritis Cartilage..

[34] Choi WS, Yang JI, Kim W (2019). Critical role for arginase II in osteoarthritis pathogenesis. Ann Rheum Dis.

[35] Zhang W, Sun G, Likhodii S (2016). Metabolomic analysis of human plasma reveals that arginine is depleted in knee osteoarthritis patients. Osteoarthritis Cartilage..

[36] Panahi Y, Alishiri GH, Parvin S, Sahebkar A (2016). Mitigation of systemic oxidative stress by curcuminoids in osteoarthritis: results of a randomized controlled trial. J Diet Suppl.

[37] Tsai WY, Wu JL, Liu CC (2013). Early intraarticular injection of hyaluronic acid attenuates osteoarthritis progression in anterior cruciate ligament-transected rats. Connect Tissue Res.

[38] Xu Z, Chen T, Luo J, Ding S, Gao S, Zhang J (2017). Cartilaginous metabolomic study reveals potential mechanisms of osteophyte formation in osteoarthritis. J Proteome Res.

[39] Zhang W, Likhodii S, Zhang Y (2014). Classification of osteoarthritis phenotypes by metabolomics analysis. BMJ Open.

[40] Zhai G, Wang-Sattler R, Hart DJ (2010). Serum branched-chain amino acid to histidine ratio: a novel metabolomic biomarker of knee osteoarthritis. Ann Rheum Dis.

[41] Bassit RA, Sawada LA, Bacurau RFP, Navarro F, Rosa L (2000). The effect of BCAA supplementation upon the immune response of triathletes. Med Sci Sports Exerc.

[42] Yang X, Yang ZJ, Liu FX (2013). Inhibition of mTOR and HIF pathways diminishes chondro-osteogenesis and cell proliferation in chondroblastoma. Tumor Biol.

[43] Davidson EB, Vitters E, Bennink M (2015). Inducible chondrocyte-specific overexpression of BMP2 in young mice results in severe aggravation of osteophyte formation in experimental OA without altering cartilage damage. Ann Rheum Dis.

[44] Kakazu E, Kanno N, Ueno Y, Shimosegawa T (2007). Extracellular branched-chain amino acids, especially valine, regulate maturation and function of monocyte-derived dendritic cells. J Immunol.

[45] Appuhamy JRN, Knoebel NA, Nayananjalie WD, Escobar J, Hanigan MD (2012). Isoleucine and leucine independently regulate mTOR signaling and protein synthesis in MAC-T cells and bovine mammary tissue slices. J Nutr.

[46] Vasheghani F, Zhang Y, Li YH (2015). PPARγ deficiency results in severe, accelerated osteoarthritis associated with aberrant mTOR signalling in the articular cartilage. Ann Rheum Dis.

[47] Chicoine LG, Paffett ML, Young TL, Nelin LD (2004). Arginase inhibition increases nitric oxide production in bovine pulmonary arterial endothelial cells. Am J Physiol Lung Cell Mol Physiol.

[48] Pietkiewicz J, Bryła J (1996). Comparison of influence of 2-oxoglutarate and glutamate on arginase activities in liver and kidney-cortex of rabbit, Oryctolagus cuniculus. Comp Biochem Physiol B.

